# Rapid Quantification of Microalgae Growth with Hyperspectral Camera and Vegetation Indices

**DOI:** 10.3390/plants10020341

**Published:** 2021-02-10

**Authors:** Pauliina Salmi, Matti A. Eskelinen, Matti T. Leppänen, Ilkka Pölönen

**Affiliations:** 1 Spectral Imaging Laboratory, Faculty of Information Technology, P.O. Box 35, FI-40014 Jyväskylä, Finland; matti.a.eskelinen@gmail.com (M.A.E.); ilkka.polonen@jyu.fi (I.P.); 2 Laboratory Centre, Finnish Environment Institute, Survontie 9A, FI-40500 Jyväskylä, Finland; matti.t.leppanen@ymparisto.fi

**Keywords:** mobile spectral camera, vegetation indices, monitoring, transmission imaging

## Abstract

Spectral cameras are traditionally used in remote sensing of microalgae, but increasingly also in laboratory-scale applications, to study and monitor algae biomass in cultures. Practical and cost-efficient protocols for collecting and analyzing hyperspectral data are currently needed. The purpose of this study was to test a commercial, easy-to-use hyperspectral camera to monitor the growth of different algae strains in liquid samples. Indices calculated from wavebands from transmission imaging were compared against algae abundance and wet biomass obtained from an electronic cell counter, chlorophyll *a* concentration, and chlorophyll fluorescence. A ratio of selected wavebands containing near-infrared and red turned out to be a powerful index because it was simple to calculate and interpret, yet it yielded strong correlations to abundances strain-specifically (0.85 < *r* < 0.96, *p* < 0.001). When all the indices formulated as A/B, A/(A + B) or (A − B)/(A + B), where A and B were wavebands of the spectral camera, were scrutinized, good correlations were found amongst them for biomass of each strain (0.66 < *r* < 0.98, *p* < 0.001). Comparison of near-infrared/red index to chlorophyll *a* concentration demonstrated that small-celled strains had higher chlorophyll absorbance compared to strains with larger cells. The comparison of spectral imaging to chlorophyll fluorescence was done for one strain of green algae and yielded strong correlations (near-infrared/red, *r =* 0.97, *p* < 0.001). Consequently, we described a simple imaging setup and information extraction based on vegetation indices that could be used to monitor algae cultures.

## 1. Introduction

Spectral cameras are used for remote sensing of algae biomass in oceans, coastal areas, and lakes [1,2], and perhaps less frequently, but to an increasing extent, to monitor cultured algae [3]. A spectral camera detects the electromagnetic spectrum of imaged material. Instead of a plain two-dimensional image, a spectral camera produces a dataset, also called a data cube, in which each pixel on the imaged surface contains information about its reflectance or transmittance across a variety of wavebands [4,5]. Hyperspectral typically refers to a system that collects data from hundreds of wavebands. Due to their practical size and versatility, spectral cameras could enable a range of algae-related applications, in addition to remote sensing.

Algae are interesting targets for spectral imaging, because they have inherent bio-optical properties that enable their optical detection. Vegetation indices are a typical method to correlate the occurrence of algae and spectral data in remote sensing [6,7], but those could be applied in a variety of scales [8,9]. At their simplest, vegetation indices are ratios of two wavebands, indicating the presence of photosynthetic pigments, that are considered as a measure of algae biomass. For example, ratios of A/B, where A was a near-infrared (NIR) waveband and B was a red waveband, have been correlated to chlorophyll *a* concentration [7]. Other typically applied index types are A/(A + B) and (A − B)/(A + B), where A and B are wavebands of a spectral camera [6,9]. The platforms and instrumentations vary, having different wavebands available. For this reason, congruence between indices and the ground variable of interest, such as algae biomass, typically needs to be established case-specifically [6,7].

Previous studies of spectroscopic monitoring of algae cultures include the use of spectroradiometers [10], and RGB cameras [3,11]. Research on hyperspectral cameras for algal determination has focused specifically on data processing and algorithm development [3]. Polerecky et al. [12] developed a hyperspectral imaging system that could be applied in various volumetric scales. The main motives and research questions that they proposed were related to natural microbial ecology and microbial communities. Mehrubeoglu et al. [13] imaged microalgae cultures in pairwise mixtures to assess their proportions using constrained linear spectral unmixing. Li et al. [14] tested different data processing methods and models to resolve lipid concentration of *Scenedesmus obliquus* from near-infrared transmission spectral images. Although spectral cameras are increasingly used, their usability might still be restrained by their high price and the complexity of data processing [3].

The purpose of this study was to compile a simple imaging arrangement based on a small SpecimIQ spectral camera [15], and test it with different control methods to quantify microalgae in cultures. This investigation consisted of two experiments. In the first experiment (hereafter Experiment I), we cultured five different algae strains belonging to cyanobacteria (*Microcystis* sp. and *Synechococcus* sp.), cryptophytes (*Cryptomonas ovata*), dinoflagellates (*Peridinium cinctum*), and green algae (*Desmodemus maximus*), and compared indices calculated from spectral data to algae abundances and biomasses assessed with electronic cell counter and chlorophyll *a* concentration assessed with an ethanol extraction method. In the second experiment (Experiment II), we compared indices calculated from spectral data to standard single-channel fluorometry when quantifying green algae (*Raphodocelis subcapitata*) in an ISO 8692:2012 standard toxicity test. The culturing in Experiment I was repeated twice and in Experiment II three times. Cultures were imaged on 24 well plates (Experiment I) or 96 well plates (Experiment II). In both experiments, we scrutinized the ratio of NIR/Red. For the red waveband, we chose a 676 nm waveband (7 nm FWHM), because it showed the lowest transmittance on the chlorophyll red absorbance area. For the reference (NIR) waveband, we chose 751 nm (7 nm FWHM) as conforming Serodio et al. [9]. However, we also calculated all possible indices formulated as A/B, A/(A + B), and (A − B)/A + B), where A and B are wavebands of the spectral camera, to observe which wavebands correlate the most strongly. For readability, the tested algae strains are referred to by their genus name throughout the text.

## 2. Results and Discussion

### 2.1. Cell Abundance and Near-Infrared (NIR)/Red Ratio

**Figure 1:**
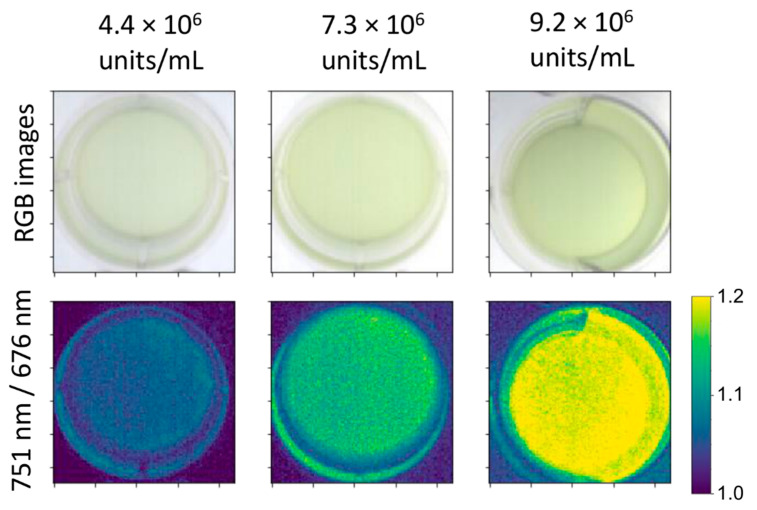
Example images of *Microcystis* culture at three different cell concentrations. The upper panels show RGB images, and the lower panels are maps where the near-infrared (NIR)/Red ratio is displayed in each pixel.

In Experiment I, cell abundances at the start of the culturings varied from 0.6 × 10^6^ cells mL^−1^ to 1.8 × 10^6^ cells mL^−1^ for *Microcystis* and from 7.0 × 10^6^ cells mL^−1^ to 8.4 × 10^6^ cells mL^−1^ for *Synechococcus.* Abundances of other strains varied between 0.2–1.9 × 10^4^ cells mL^−1^. Cultures were monitored in their exponential growth phase. Change in algae abundance between time points was typically minor for the naked eye; however, differences could be displayed based on the spectral data (Figure 1), an advantage of an imaging system, as also demonstrated by Polerecky et al. [12] and Li et al. [14]. For processing, a rectangle ROI (Region of Interest) that excluded the edges of the sample well was chosen to make data processing as straightforward as possible.

**Figure 2:**
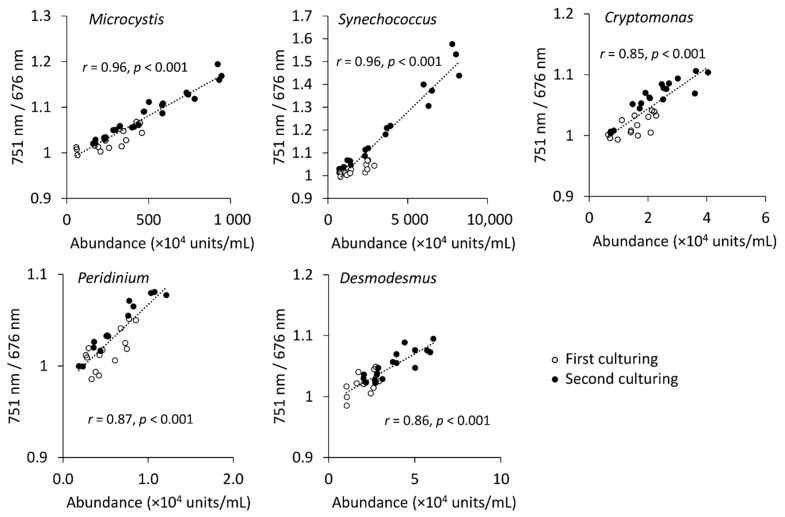
Correlation between algae abundances assessed with cell counter and the ratio of 751 nm and 676 nm wavebands.

**Figure 3:**
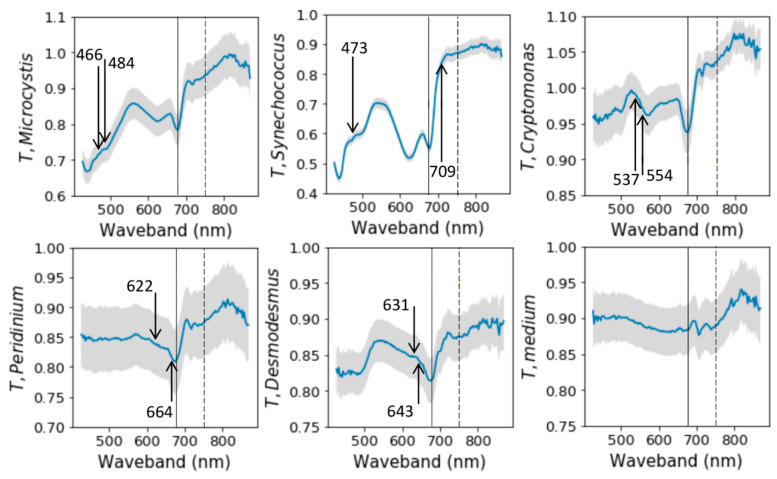
Examples of transmittance (*T*) spectra of each strain and the culture medium from the last sampling date. Blue lines describe mean spectra across ROI (Region of Interest). Grey areas show the standard deviation across the ROI. Solid vertical lines show the position of 676 nm and the dashed vertical lines position of 751 nm wavebands. Arrows mark the wavebands whose ratio has the strongest correlations with algae biomass (see Table 1). Y-axes were scaled differently to facilitate the visualization of variation.

The correlations between increasing abundance and the ratio of 751 nm and 676 nm wavebands calculated as the mean of the rectangular ROI were very strong for the cyanobacteria (*r* > 0.95, *Microcystis* and *Synechococcus*), and strong (0.85 ≤ *r* ≤ 0.87) for the other tested strains (Figure 2 and Figure 3). The lowest algae abundances had the ratio of 751 nm/676 nm calculated from mean spectra lower than 1, which might indicate that the algae abundances were below the detection limit of the spectral camera. When *Microcystis* reached approximately 2 × 10^6^ cells mL^−1^ and *Synechococcus* 8 × 10^6^ cells mL^−1^, the ratio of the two wavebands stayed above 1. The same was observed for *Cryptomonas* at 2 × 10^4^ cells mL^−1^, *Peridinium* at 6 × 10^3^ cells mL^−1^ and *Desmodesmus* at 2 × 10^4^ cells mL^−1^. The correlations between abundances and the ratio of 751 nm/676 nm wavebands calculated from the mean transmittance spectra showed that this spectral imaging method could be applied for the robust monitoring of algae growth.

### 2.2. Biomass and NIR/Red Ratio

**Figure 4:**
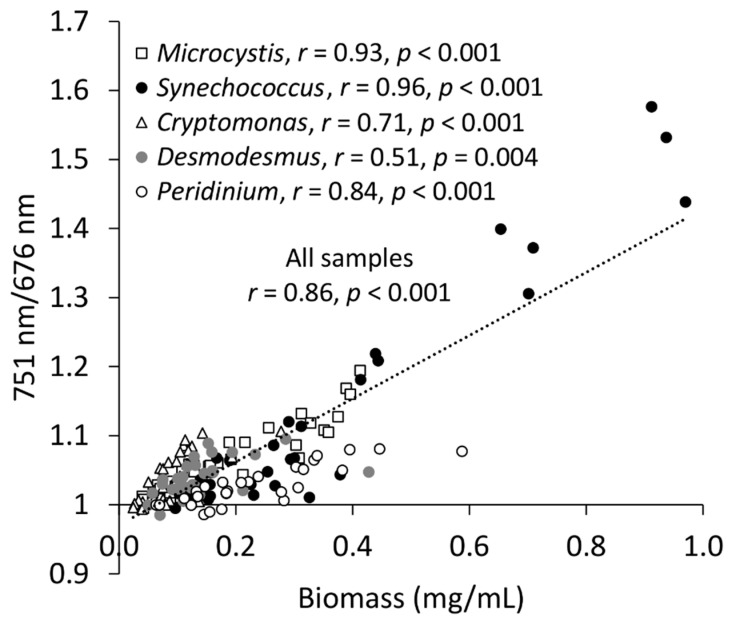
Correlation between algae biomass assessed with cell counter and ratio of 751 nm and 676 nm wavebands on the mean transmittance spectra.

As the cell size of algae generally varies greatly, algae biomass might be the most expedient metric for comparisons in algae monitoring and research. When the spectral indices were compared to biomass estimates, results from the cultures could be drawn in the same figure (Figure 4), which was impossible for the abundance results with differences up to several orders of magnitude. The general overview was that the correlation between biomass and 751 nm/676 nm was strong (*r* = 0.86, *p* < 0.001, Figure 4). However, biomass assessments introduced variation into the correlations. *Desmodemus* had only a fair correlation (*r* = 0.51, *p* = 0.004) between biomass and the spectral index (Figure 4). This was likely due to the occurrence of aggregated coenobia in the cell counter that increased the variation in biomass estimates, but not so much in abundance assessments (Figure 2). If one sample with a notable *Desmodesmus* aggregation, was omitted, the correlation between *Desmodesmus* biomass and 751 nm/676 nm improved (*r* = 0.69, *p =* 0.004). For the other, generally single-celled strains, correlations were either moderate (*r* = 0.71, *p* < 0.001, *Cryptomonas*) or strong (*r* = 0.93 for *Microcystis*, *r* = 0.96 for *Synechococcus*, *r* = 0.84 for *Peridinium*, *p* < 0.001 for all). The correlation between biomass and the spectral index of all the strains was generally strong (*r* = 0.86, *p* < 0.001, Figure 3), due to the high biomasses of fast-growing *Synechococcus*. Separate plots of each strain are included in the Supplementary Materials (Figure S1).

### 2.3. Chlorophyll a Concentration and NIR/Red Ratio

**Figure 5:**
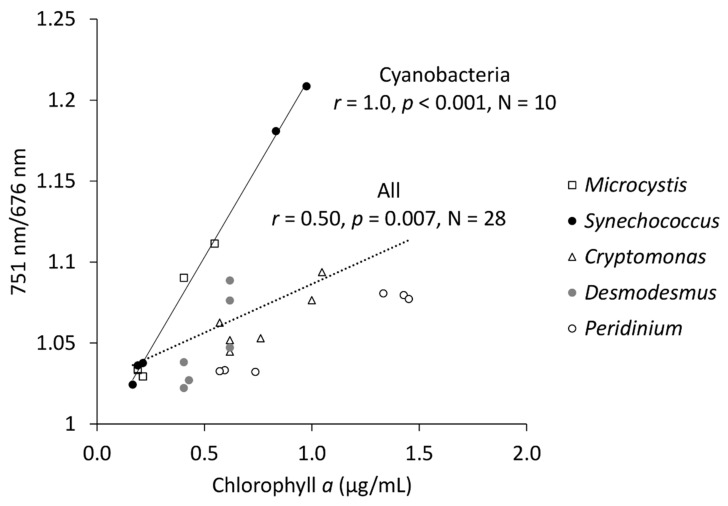
Correlation between chlorophyll concentration and the ratio of 751 nm and 676 nm wavebands. N—number of samples.

Aggregation of pigments in cells and aggregation of cells affects the transmittance of light through an algae suspension. This package effect [16,17] means that given the same total concentration of chlorophyll *a*, more densely packed and larger aggregates enable higher transmittance compared to loosely packed and smaller cells. In our experiment, *Microcystis* and *Synechococcus* had a higher 751 nm/676 nm spectral index with lower chlorophyll *a* concentration (Figure 5). These two strains were small-celled and highly abundant, whereas the other strains had larger cell-sizes, but notably lower abundances. At the time of this comparison, the mean cell diameter for *Microcystis* according to the cell counter was 3.9 µm (*SD* = 0.04) and for *Synechococcus* 2.5 µm (*SD* = 0.02). The cell diameter for *Cryptomonas* was 18.7 (*SD* = 0.45) and for *Desmodesmus* 17.6 µm (*SD* = 0.53). *Peridinium* had a cell diameter of 40.3 µm (*SD* = 0.67). For the cyanobacteria, the correlation between chlorophyll *a* and 751 nm/676 nm was perfect (*r* = 1.00, *p* < 0.001, Figure 5); however, when all the sampled strains were scrutinized together, the correlation was only fair (*r* = 0.50, *p* = 0.007), likely due to the differences caused by the package effect. This was also observed when the index was compared against wet biomass (Figure 4), because *Peridinium* yielded notably lower indices with relatively high biomasses. However, in the biomass comparison (Figure 4), this effect was not as conspicuous as in chlorophyll *a* comparison (Figure 5).

### 2.4. Chlorophyll Fluorescence

**Figure 6:**
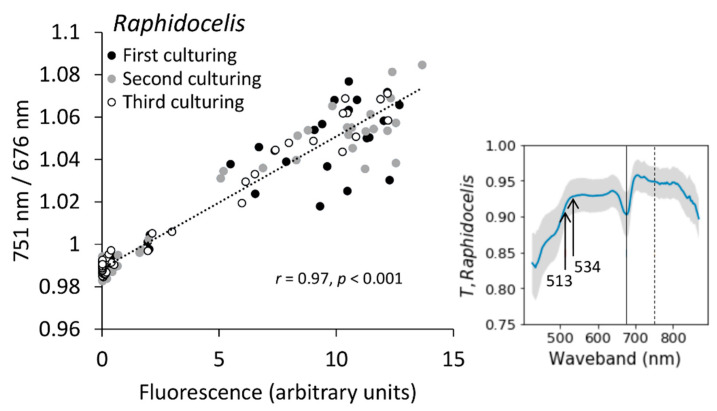
Correlation between chlorophyll fluorescence and ratio of 751 nm and 676 nm wavebands (**left**). Example transmittance (*T*) spectrum of *Raphidocelis* from the last culturing day (**right**). Solid vertical lines show the position of 676 nm and dashed vertical lines position of 751 nm wavebands. Arrows mark the wavebands with the strongest correlation to fluorescence (see Table 1).

Generally, the ratio of NIR/Red (751 nm/676 nm) correlated well with chlorophyll fluorescence measured with a bench-top fluorometer (*r* = 0.97, Figure 6). However, especially the second and third culturing experiments had some variation (Figure 6). Possible reasons might be biological (algae cells displaying variety in fluorescence) or technical (e.g., position of the samples in the image). Here, the ROI was only 20 × 20 pixels compared to 50 × 50 pixels of the image processing done for the five strains reported above, because we used 96-well plates for imaging instead of the 24-well plates that were used in Experiment I. The results from Experiment II indicated that transmittance-based assessment could be practical, and likely an option for toxins or concentrations that hamper biomass estimations based on fluorescence [18].

### 2.5. Indices with the Strongest Correlations

Above, we presented results based on an index of the selected wavebands (751 nm and 675 nm) in Experiment I and II. However, our spectral camera recorded 204 different wavebands with equal intervals, enabling us to test which waveband ratios had the strongest correlations to algae biomass. The outcome was that the index yielding the highest correlation to biomasses was different for each tested strain (Table 1), and none of the strongest correlations was based on the NIR/Red ratio tested above. This means that the studied algae might also have other spectral features that could indicate their growth more precisely than the NIR/Red ratio did.

*Synechococcus* correlated best on red/blue waveband and *Microcystis* on green/blue (Figure 3, Table 1). In contrast, *Cryptomonas* had the strongest correlation on the green wavebands, which might be explained by phycobiliproteins that are typical to cryptophytes [19]. *Peridinium* and *Desmodesmus* had the strongest correlations on red wavebands.

Choosing the wavebands accordingly for an algae strain of interest might yield a more precise indication of changes in the culture. However, when choosing the index, it is important to understand the features (e.g., dominant pigments) explaining the variation. These features need to be identified depending on the target of monitoring or research, but were not a focus of this study.

## 3. Conclusions

The simple method based on commercial, easy-to-use spectral camera, and vegetation indices was suitable for robust monitoring of algae growth before the stationary growth phase. Compared to the other established methods, the advantages of the method described here are visual documentation, a non-destructive way of measurement, and the possibility to move the camera to establish different imaging setups in varying volumetric scales (two different tested here). The use of NIR/Red waveband ratios yielded strong correlations to algae abundances (0.85 < *r* < 0.96, *p* < 0.001) and generally strong or moderate to biomasses (0.51 < *r* < 0.93, *p* < 0.001). The comparison of spectral imaging to chlorophyll fluorescence was done for one strain of green algae and yielded strong correlations (NIR/Red, *r =* 0.97, *p* < 0.001). For each strain, amongst indices formulated as A/B, A/(A + B) or (A − B)/(A + B), where A and B were wavebands of the spectral camera, good correlations to biomass were found (0.66 < *r* < 0.98, *p* < 0.001). No universal index was found that could correlate to algae biomass for all the species equally well, likely due to varying optical features, such as cell size and pigment compositions. This method is, therefore, best suited for monitoring pure cultures, perhaps those with pigments of commercial interest.

## 4. Materials and Methods

This study consisted of two separate experiments (I and II). In Experiment I, three replicates of five different algae strains were cultured twice. Experiment II was committed alongside an algae fluorescence-based standard toxicity test (ISO 8692:2012) that was repeated by three separate culturings. Each culturing in Experiment II consisted of 30 samples with algae exposed to varying concentrations of potassium dichromate, 10 samples of potassium dichromate solution, 11 samples of culture media or water, and 9 samples of algae culture with no exposures.

### 4.1. Hyperspectral Imaging System

Algae samples on clear, flat-bottom 24-well or 96-well plates were imaged in transmission light using a SpecimIQ mobile spectral camera (Specim, Oulu, Finland). SpecimIQ has a wavelength band of 400–1000 nm and a CMOS sensor with 204 spectral bands (7 nm FWHM). The camera has been described in more detail in [15]. Our light source was a broadband halogen (Fiber-Lite, DC-950, Dolan-Jenner, Boxborough, MA, USA) with a diffusor plate (Dolan-Jenner, USA). A well plate including the algae samples was placed on the diffusor. The distance between the diffusor and the scanner’s lens was 14–14.5 cm when comparing the spectral camera with cell counter assessment (Experiment I) and 12 or 14 cm when comparing spectral camera with fluorometry (Experiment II). Spatial pixel size was determined by measuring the width of a sample well each imaging day. The pixel width was, on average, 171 µm (*SD* = 8) in Experiment I and 164 µm (*SD* = 13) in Experiment II. Imaging was done using 21 ms (first culturing of Experiment I and all of Experiment II) or 12 ms (second culturing of Experiment I) integration time. Imaging was done in a dark room (Experiment I) or in dim room light (Experiment II). In both experiments, the imaging setup was covered with a light impermeable hood during imaging.

**Figure 7:**
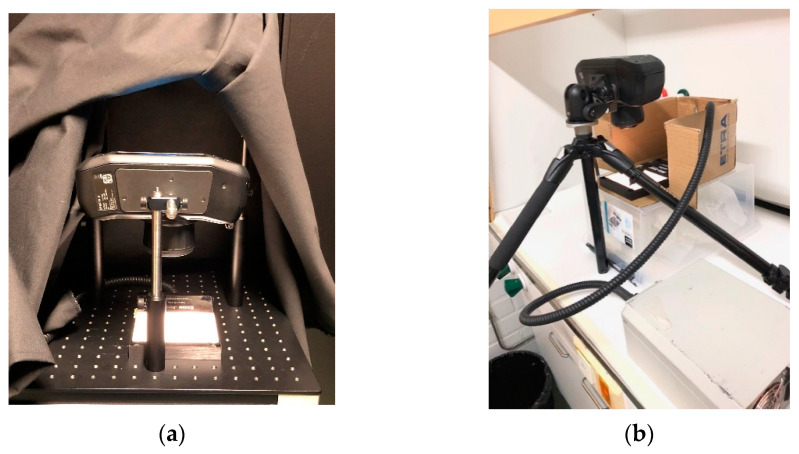
SpecimIQ imaging arrangement in (**a**) Experiment I—comparison of spectral imaging and electronic cell counter and (**b**) Experiment II—comparison of spectral imaging and chlorophyll fluorometry.

Experiment I was done using 24-well plates and the arrangement, shown in Figure 7a. In Experiment II, the imaging was done in a fume hood using 96-well plates covered with a transparent lid (Figure 7b). An average spectrum of the diffusor plate without the sample plate was used as a white reference that was determined again before each imaging session. In Experiment II, we aimed to overcome slight spatial fluctuations on the diffusor’s light field even further, and instead of average, we normalized the raw sample images using a raw image of the light source. In both experiments, we used SpecimIQ’s pre-programmed value as the dark reference.

### 4.2. Experiment I

This experiment consisted of 27 separate imaging sessions yielding 166 imaged samples of pure cultures. Algae strains of this first experiment were from the Culture Collection of Algae at the University of Cologne (CCAC). The following pure cultures were used: CCAC 3504 B *Microcystis* sp., CCAC 2944 B *Synechococcus* sp., CCAC 0064 *Cryptomonas ovata*, CCAC 0102 B *Peridinium cinctum*, and CCAC 3524 B *Desmodesmus maximus*. All strains were of freshwater origin. To facilitate reading, the strains are referred to by their genus names.

Three replicates of each strain were cultured in Waris-H + 3V + Si medium [20]. The algae were cultured at +15 °C temperature in 250 mL tissue culturing flasks with filter caps (VWR International). Light intensity was adjusted to 7.9–8.8 kLux at the level of the flask caps using four fluorescent lamps installed with a dimmer (two Phillips TLD 36W/950 and two Aura 36W 658, alternately). A light:dark cycle of 14:10 h was used. Here, we aimed for the rather slow growth of the algae so that we could scrutinize the sensitivity of the imaging system.

The duration of the first culturing was five weeks (35 days), during which each replicate was sampled at least once a week for spectral imaging so that the total number of sampling times was four for *Desmodesmus* and five for the other strains. The last samples of this culturing were taken after the algae had been at least 1 day, and a maximum of 5 days, in darkness, due to an electrical malfunction in the culturing facilities. Although this shortcoming might have increased variation in the spectral features of the algae, we consider that it did not compromise the capability of our experimental design to respond to our research aims. New similar culturings were established and cultured again for 7 weeks (*Microcystis* and *Synechococcus*), 6 weeks (*Cryptomonas* and *Desmodemus*), or 5 weeks (*Peridinium*) using a similar setup as in the first culturing.

Cultures were handled aseptically when samples were taken for imaging. To mix the cultures, air bubbles were blown with an electronic pipette with sterile filter tips for ten seconds. A sample volume of 2 mL was transferred to a clear flat-bottom 24-well plate (Sarstedt). These wells were used first for transmission spectral imaging (Figure 7a) and immediately after that for reference analysis with an electronic cell counter (Casy, Omni Life Sciences, Bremen, Germany). The counting results were processed with CASYworkX 1.26 macro (Omni Life Sciences) for Microsoft Excel. The Casy electronic cell counter records the number of particles, and assesses particle biovolume based on pulse area analysis of the measuring signal. A measurement capillary for the counter was chosen according to the cell or coenobia sizes of the strains; a 60 µm capillary was chosen for cyanobacteria and a 150 µm capillary for other tested strains. Dilution was adjusted according to the algae abundance; a 10–200 µL sample was suspended in 10 mL of CasyTon buffer for cyanobacteria and a 200–1000 µL sample in 10 mL of CasyTon buffer for other tested strains. The lower limit for particles counted as algae (left evaluation cursor) was set at 2 µm for *Microcystis*. For *Synechococcus,* no lower limit was set due to the small cell diameter of the strain. For *Cryptomonas* and *Desmodesmus,* the lower limits were set at 10 µm, and at 29 µm for *Peridinium*. Particle size distribution, yielded by the electronic cell counter and preliminary knowledge of the strain morphology, was used as a basis for setting the lower limits (Figures S2 and S3). Algae biovolumes in the samples, obtained with the electronic cell counter (fL/mL), were converted to wet biomass (mg/mL) by assuming the cells to be isopycnic to water.

During the second culturing, samples for chlorophyll *a* concentration assessment were taken for the first time at an early state of the culturing and a second time 2 to 4 weeks later. Samples (5 mL) were taken at the conjunction of the samplings for the other reference methods and handled in dim light and sheltered from light using aluminum foil. Samples were filtered gently on GF/C filters (Whatmann, Maidstone, UK). Filters were stored wrapped in 15 mL tubes (Nunc) at −20 °C for 2 to 3 months before pigments were extracted using hot 94% ethanol (w%) extraction. In this method, filters were immersed in 10 mL ethanol and incubated at 75 °C for 5 min. Samples were clarified by filtering them through 0.45 µm pore size nylon syringe filters (Whatman, Uniflo 25 mm) directly into a 1 cm quartz kyvette. Absorbance was measured with a Shimadzu UV-1800 spectrophotometer on 665 nm and 750 nm wavebands. The concentration of chlorophyll *a* in a sample was calculated using an absorption factor of 11.9 according to SFS-ISO 10260:1992.

### 4.3. Experiment II

The experimental setup of this second experiment was repeated three times, yielding 180 imaged and fluorometrically measured samples. Green algae (*Raphidocelis subcapitata*) originating from the Culture Collection of Algae at the University of Göttingen (SAG) were acclimated and enriched in a freshwater medium (Finnish Environment Institute, pH 8) at 23 ± 2 °C at approximately 10 kLux fluorescent light for four days. After acclimation, diluted inocula of algae were cultured on 96-well plates for 72 h in the same conditions as during acclimation. The algae were exposed to potassium dichromate, which is used as a reference toxin when testing growth inhibition conforming to ISO 8692:2012. Potassium dichromate was added as a dilution series with 0.25 mg L^−1^ increments from a concentration of 0.5 mg L^−1^ to 2.75 mg L^−1^. At the beginning of the experiment, the cell abundance was approximately 8 × 10^3^ cells mL^−1^ in each well, and the sample volume in each well was 300 µL. Abundance was confirmed by microscoping the acclimated inoculumn with a hemocytometer. After 72 h of culturing, chlorophyll autofluorescence was measured with a plate reader (excitation 450 nm, emission 680 nm, Varioskan Flash, Thermo Fisher Scientific, Waltham, MA, USA). Background for the potassium dichromate, ultrapure water, and inoculumn unexposed to potassium dichromate were also treated as potassium dichromate exposed samples and measured accordingly. Transmission spectral images of the well plates were taken immediately after the fluorometric measurements with the setup, shown in Figure 7b.

### 4.4. Index Calculation

Spectral images were processed, and the ratio of NIR/Red, as well as all the indices formulated as A/B, A/(A + B), or (A − B)/(A + B), where A and B are wavebands of the spectral camera, were calculated using Python 3.7 with numerical numpy-library and visualized with matplotlib -library. From each imaged sample well, a data cube with spatial dimensions of 50 × 50 pixels (Experiment I, 24-well plates) or 20 × 20 pixels (Experiment II, 96-well plates) was extracted for further processing (Region of Interest, ROI). The imaging was done in transmission mode, but as Polerecky et al. [12] noted, transmission data normalized to illumination can be treated similarly to reflectance data.

## Figures and Tables

**Table 1:** Wavebands with strongest correlations for algae biomass (1–5) or chlorophyll fluorescence (7) and different index types for the five tested strains when A is on NIR and B on red waveband area, *p* < 0.001 for all shown correlation coefficients. Wavebands are shown without units (nm). Colors indicate roughly position on the visual spectrum (blue-green-red).

	Alga	A/B	A/(A + B)	(A − B)/(A + B)
1	*Synechococcus*	709/473, *r* = 0.98	859/(859 + 473), *r* = 0.98	(859 − 473)/(859 + 473), *r* = 0.98
2	*Microcystis*	484/466, *r* = 0.96	537/(537 + 464), *r* = 0.96	(537 − 464)/(537 + 464), *r* = 0.96
3	*Cryptomonas*	537/554, *r* = 0.79	537/(537 + 554), *r* = 0.79	(537 − 554)/(537 + 554), *r* = 0.79
4	*Peridinium*	622/664, *r* = 0.92	622/(622 + 664), *r* = 0.92	(622 − 664)/(622 + 664), *r* = 0.92
5	*Desmodesmus*	631/643, *r* = 0.66	631/(631 + 643), *r* = 0.65	(631 − 643)/(631 + 643), *r* = 0.65
6	*Raphidocelis*, *fluorescence*	513/534, *r* = 0.98	513/(513 + 534), *r* = 0.98	(513 − 534)/(513 + 534), *r* = 0.98

## Data Availability

The data generated in this study is stored in the JYX repository at the URI: http://urn.fi/URN:NBN:fi:jyu-202009035740 (accessed on 13 January 2021) and DOI:10.17011/jyx/dataset/71623.

## Supplementary Materials

The following are available online at https://www.mdpi.com/2223-7747/10/2/341/s1, Figure S1: Correlation between algae biomasses assessed with cell counter and the ratio of 751 nm and 676 nm wavebands, Figure S2: Microscopy images of the stains in Experiment I., Figure S3: Example graphs of particle distributions obtained by the electronic cell counter.
