# Supplementary material for: Rapid Quantification of Microalgae Growth with Hyperspectral Camera and Vegetation Indices
Source: Plants (Basel). 2021 Feb 10;10(2):341. doi: 10.3390/plants10020341 (PMC7916729; doi:10.3390/plants10020341)
Supplement: Supplementary file 1 [file plants-10-00341-s001.pdf]

Supplementary material

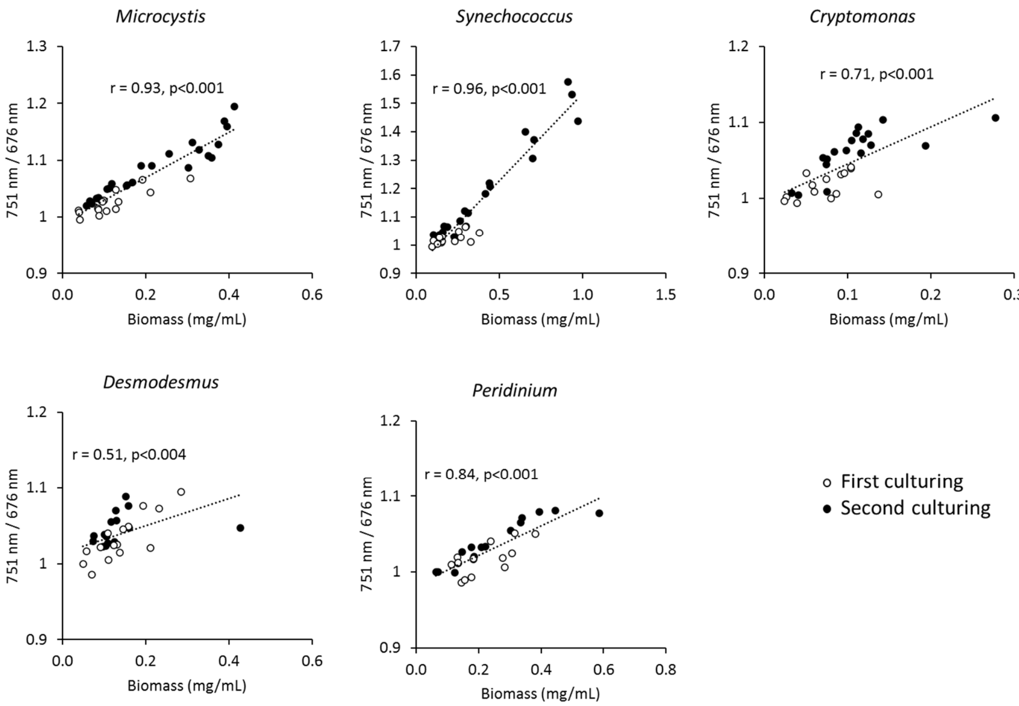

Figure S1. Correlation between algae biomasses assessed with cell counter and the ratio of 751 nm and 676 nm wavebands.

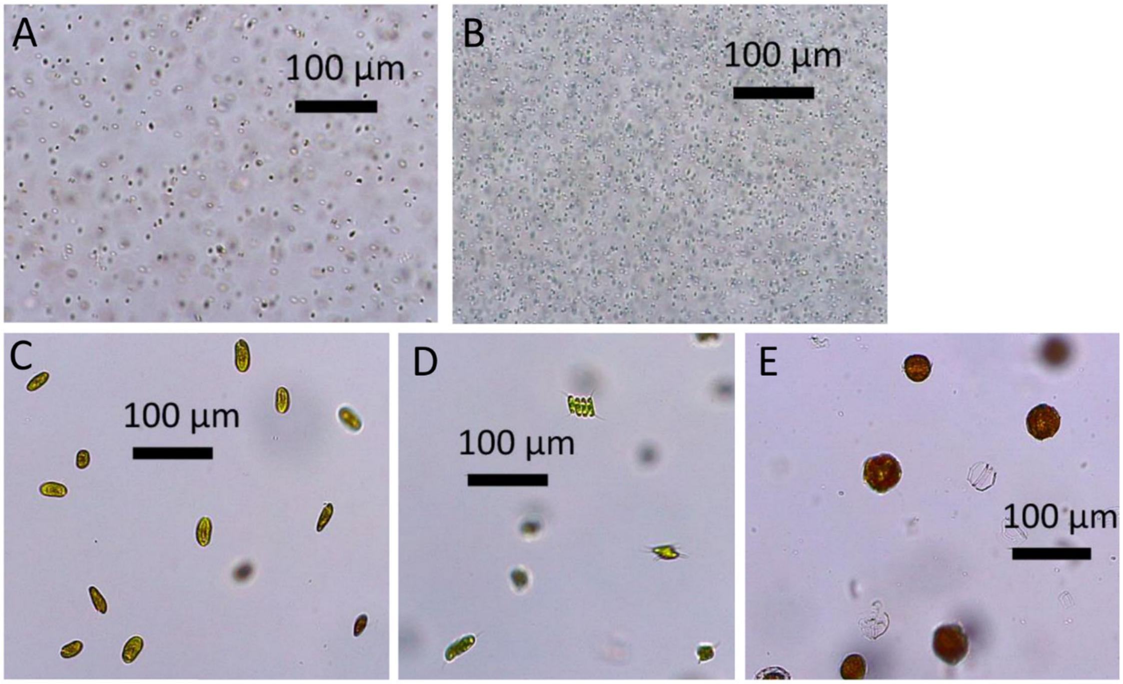

Figure S2. Microscopy images of the stains in Experiment I. A – *Microcystis*, B – *Synechococcus*, C - *Cryptomonas*, D – *Desmodesmus* and E – *Peridinium*.

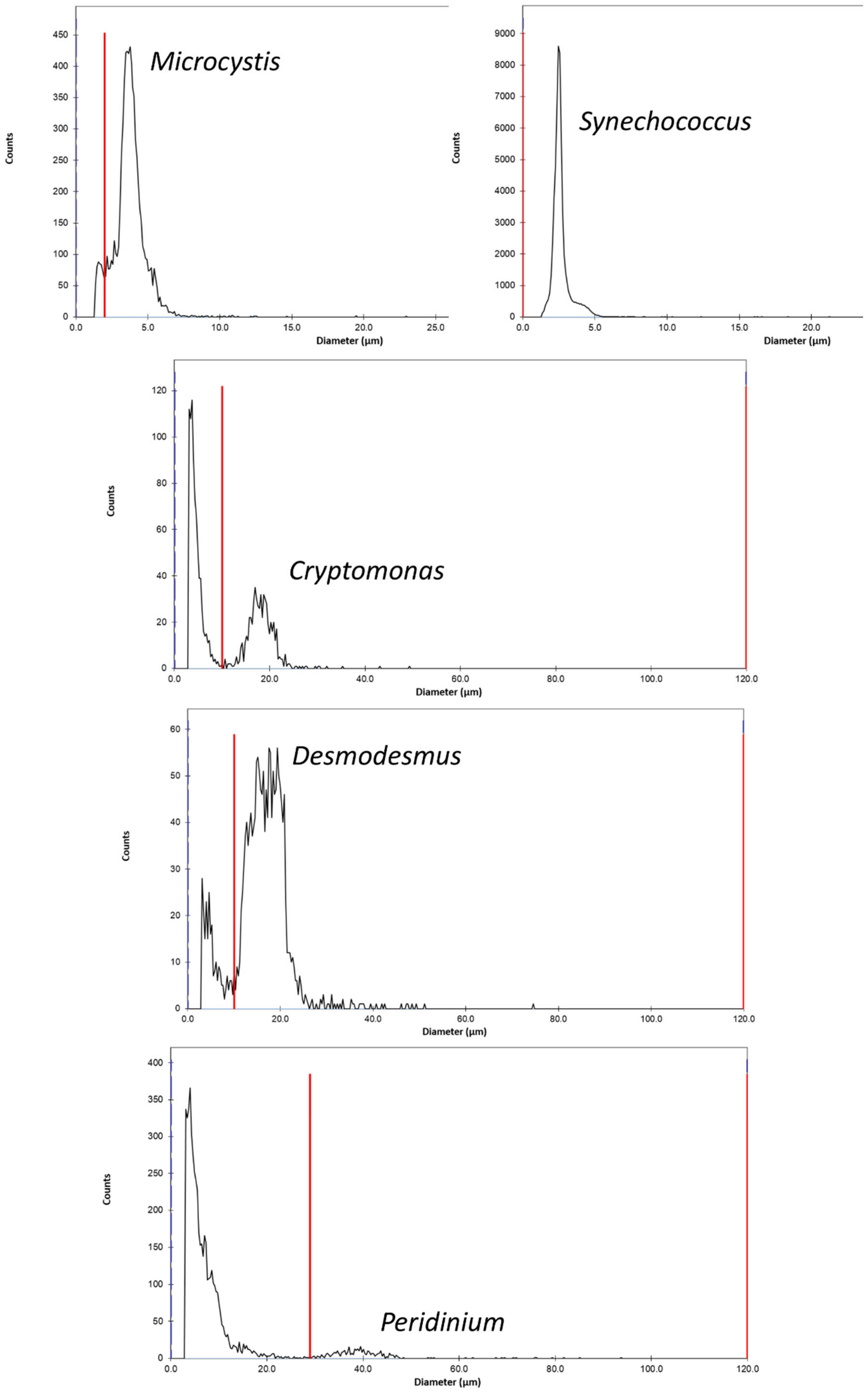
Microcystis
Synechococcus
Cryptomonas
Desmodesmus
Peridinium
Counts
Diameter (µm)

8

Figure S3. Example graphs of particle distributions obtained by the electronic cell counter. Red vertical lines represent the evaluation cursors, and all the counts with larger cell diameter than the left evaluation cursor were considered as algae. Right evaluation cursor (the upper limit) was set at the largest possible values with the chosen capillary.
